# The Wild Side of Science: Rodents and Bats in the Zoonotic Equation

**DOI:** 10.3390/v17070987

**Published:** 2025-07-16

**Authors:** Gábor Kemenesi, Kornélia Kurucz

**Affiliations:** National Laboratory of Virology, University of Pécs, H-7624 Pécs, Hungary; kurucz.kornelia@pte.hu

## 1. Introduction

Bats and rodents represent the most species-rich and widespread mammalian groups on our planet. Unsurprisingly, the diversity of viruses they carry also follows this trend. Therefore, they are known as a significant host to a wide range of viruses and are recognized as reservoirs for certain viruses with important implications for human health [1]. This makes them primary targets for surveillance studies in virology, especially for trending and potential zoonotic agents.

However, the picture is more complex, particularly in the context of zoonotic spillover events affected by global changes that are either widely known or currently under investigation. These global trends, such as climate change and human disturbance, are changing and entangling the patterns of human–animal encounters and thus the risk of epidemics. Due to these changing patterns, it is yet unknown how it may impact the role of rodents and bats in disease emergence in the near-future [2]. Ultimately, their distribution, evolutionary history, reservoir role, and relative position on the interaction map bring them into focus of zoonotic pathogen research.

Classical surveillance studies have long been used as essential tools for understanding virodiversity in nature. Nevertheless, the tools and scope of surveillance have rapidly evolved and diversified during recent decades. Nowadays, the focus of surveillance work can span ecological monitoring, pathogen discovery, molecular modeling, and in vitro experiments to reveal the most important aspects of virus–host interaction (e.g., receptor entry, immune responses).

Although the possibilities of surveillance studies have expanded significantly in recent years, with functional indications becoming easier to assess, besides the accumulation of genetic data, there is now a growing opportunity for applied risk analysis. The articles published in this Special Issue capture the diversity and importance of surveillance activities, focusing on two well-established viral reservoir hosts.

## 2. Novel Surveillance Data of Bat Coronaviruses

Multiple articles in this Special Issue exemplified the importance of collecting basic surveillance data on pathogens of interest. Since the severe acute respiratory syndrome coronavirus (SARS-CoV) epidemic in 2003, which caused the first significant epidemics after the millennium, coronavirus surveillance in bats and other animals has become an important tool to predict the risk of emergence. In this Special Issue, multiple articles reported novel sequences of bat coronaviruses from both the *Alpha*– and *Betacoronavirus* genera.

Researchers provide new insights on the growing diversity of bat coronaviruses by describing the finding of a novel Nobecovirus in a fruit bat (*Epomophorus wahlbergi*) from Nairobi, Kenya. The team found and partially sequenced the virus, known as NRB24, using the metagenomic sequencing of fecal samples taken from peridomestic habitats. This virus shows a distinct phylogenetic lineage within the Nobecovirus subgenus of Betacoronaviruses. Even though Nobecoviruses are not known to infect people currently, their genetic variability and near-proximity to livestock and highly populated metropolitan areas highlight the importance of continuous monitoring in quickly growing urban areas such as Nairobi. Our knowledge of coronavirus evolution and host range is expanded by these findings, which also emphasize urban wildlife–human interactions as vital hotspots for zoonotic risk assessment and early viral discovery. Additionally, a recombination study of the novel virus indicates a hypothetical scenario of coinfections and intra-host genetic exchange, which may facilitate viral genome diversification [3].

Two articles from the same author group report the first detections of Alphacoronaviruses in both cave- and tree- or crevice-dwelling bats in Portugal, revealing a broad ecological range of these viruses in this area. The initial investigation uncovered a relatively high prevalence (8.87%) of Alphacoronaviruses in cave-roosting bats, primarily detected in fecal samples, suggesting that denser bat populations in caves may facilitate viral maintenance. In contrast, the follow-up study in less densely populated, tree- and crevice-dwelling bat habitats found only a single positive case (1.15%), yet the virus shared striking phylogenetic similarity with strains from the same bat species across Europe, pointing to potential host specificity. Taken together, these findings further highlight the importance of habitat type, host ecology, and population density in shaping viral transmission dynamics. They also highlight the need for sustained, habitat-inclusive viral surveillance to better understand coronavirus evolution and assess zoonotic risks [4,5].

## 3. Novel Surveillance Data of Filoviruses

Filovirus research has a long and well-established history, particularly driven by the high-impact human outbreaks caused by Ebola and Marburg viruses. These two pathogens have shaped our scientific and public health understanding of viral hemorrhagic fevers for decades. However, in recent years, this once narrowly defined viral family has expanded rapidly, with a remarkable number of newly identified members emerging both within and beyond the African continent. Even when focusing solely on bat-derived filoviruses, the level of genetic and ecological diversity uncovered is reshaping our understanding of their evolution, host range, and potential for spillover.

In Vietnam, a large-scale molecular and serological survey identified filoviral RNA in *Rousettus leschenaultii* and *R. amplexicaudatus* bats, with some viral sequences clustering with the Dianlovirus genus, while others formed a distinct clade closer to Orthomarburgvirus. Serological results also indicated previous exposure in multiple fruit bat species, suggesting a broader circulation of these viruses across the region. Complementing this, a separate study documented the first detection of Bombali virus (BOMV) in *Mops condylurus* bats in Tanzania, extending the known range of this ebolavirus to East Africa. Despite the low viral load and lack of serological confirmation, phylogenetic analyses confirmed the close relatedness to Kenyan BOMV strains, reinforcing *M. condylurus* as a likely reservoir [6,7].

## 4. Novel Surveillance Data of Sosuga Virus

A comprehensive ecological investigation has confirmed the presence of Sosuga virus (SOSV), a zoonotic paramyxovirus, in Egyptian rousette bats (*Rousettus aegyptiacus*) in Sierra Leone, marking the first detection of this virus in West Africa and a significant range extension from prior detections in Uganda. Out of 377 bats sampled, 26% tested positive for SOSV RNA, with viral material found across multiple tissues, and 38% showed SOSV-specific antibodies, suggesting active circulation and population-level exposure. Notably, juvenile bats exhibited higher rates of active infection, while adults, particularly females, showed greater seroprevalence, suggesting age-dependent infection dynamics and possible sex-based differences in immune response or exposure. Despite unsuccessful virus isolation attempts, nearly complete genome sequencing and phylogenetic analyses confirmed a close genetic relationship (≥99% similarity) between the Sierra Leone strain and previously identified SOSV strains from Uganda, including one from a human case. These findings support the role of *R. aegyptiacus* as a competent reservoir for SOSV and reinforce the species’ known reservoir status for other high-consequence pathogens like Marburg virus. The study highlights the importance of continued bat surveillance in under-sampled regions to better map the ecological distribution of emerging zoonoses and inform public health risk assessments before spillover events occur [8].

## 5. The Applied Side of Surveillance Data

The coronavirus study by Li and Tahiri analyzes the cophylogenetic patterns between bat coronaviruses and their hosts across 15 geographic regions with multiple in silico methods. Notably, their analysis suggests that while overall virus–host co-speciation is limited, specific gene regions such as ORF1ab and the spike protein show stronger phylogenetic congruence with host lineages. These findings present novel data about the evolutionary plasticity of coronaviruses and point toward modular adaptation as a key mechanism facilitating host shifts and viral diversification [9].

Mello and colleagues present a spatiotemporal modeling approach of hantavirus spread in Brazil, integrating rodent host abundance and ecological suitability with SIR-cellular automata models. Both studies emphasize the value of integrating host–pathogen data into comprehensive analyses to generate novel insights into viral evolution, ecological dynamics, and spillover potential. Together, these articles highlight the critical role of predictive modeling and phylogenomic mapping as important complementary tools for understanding zoonotic spillover events and unraveling the evolutionary mechanisms that may trigger emergence or outbreaks. In doing so, they strengthen the rationale for One Health approaches that integrate field ecology, molecular evolution, and public health readiness [10].

## 6. Conclusions

The cyclical occurrence of panic and subsequent neglect in the funding of global pandemic research appears to be even more intricate than those experienced during prior public health emergencies, particularly as it is currently exacerbated by a surge in anti-science sentiment. This situation could lead to the termination of globally crucial programs such as the USAID PREDICT program. The program has identified more than 900 unique viruses, including a novel Ebolavirus, epidemiologically significant coronaviruses, and their reservoirs [11,12,13]. Essential infrastructures for pandemic preparedness, including surveillance programs critical for timely detection and response, are undergoing substantial budget reductions merely years following the global reckoning of COVID-19. This regression not only undermines the critical lessons that were painfully acquired but also diminishes global resilience at a juncture when emerging infectious threats are perpetually escalating. Disrupting this cycle necessitates a political commitment that is firmly rooted in empirical evidence and a long-term dedication to securing public health.

Surveillance studies from a diverse array of perspectives are featured in this Special Issue. These studies provide powerful evidence of the critical role that surveillance plays in advancing our understanding, prevention, and preparedness for emerging infectious diseases, whether their risks are known at present or yet to be identified.
